# Integrating Network Pharmacology and In Vitro Experiments to Elucidate the Antiatherosclerotic Mechanisms of Qingxin Tongmai Yin

**DOI:** 10.1155/mi/9752147

**Published:** 2026-08-05

**Authors:** Yangfan Huang, Zhengshu Xia, Zhiyong Yu, Jiaying Song, Jiaqian Fang, Yaohong Song, Rui Chen

**Affiliations:** ^1^ Nanjing University of Chinese Medicine, Nanjing, Jiangsu Province, China, njucm.edu.cn; ^2^ Department of Cardiology, Taihe County People’s Hospital, Fuyang, Anhui Province, China; ^3^ Kangda College of Nanjing Medical University, Lianyungang, Jiangsu Province, China, njmu.edu.cn; ^4^ Department of Cardiology, Nanjing Hospital of Chinese Medicine Affiliated to Nanjing University of Chinese Medicine, Nanjing, Jiangsu Province, China, njucm.edu.cn

**Keywords:** AGE-RAGE signaling pathway, atherosclerosis, inflammation, network pharmacology, Qingxin Tongmai Yin decoction

## Abstract

Qingxin Tongmai Yin (QXTMY), a classic traditional Chinese medicine (TCM) formula widely used for cardiovascular and cerebrovascular diseases, has unclear multitarget mechanisms against atherosclerosis (AS). This study integrated network pharmacology with experimental validation to investigate these mechanisms. Network pharmacology identified 128 bioactive compounds and 135 potential targets of QXTMY, highlighting key anti‐AS targets such as TNF, IL6, insulin (INS), IL1B, MMP9, CCL2, and ALB, with the AGE‐RAGE signaling pathway as a central mechanism; principal active ingredients included quercetin, kaempferol, luteolin, and cryptotanshinone. In vitro experiments using THP‐1‐derived macrophages induced with phorbol‐12‐myristate‐13‐acetate (PMA) and oxidized low‐density lipoprotein (ox‐LDL) showed that QXTMY significantly suppressed inflammatory responses, inhibited foam cell formation, and downregulated the AGE‐RAGE signaling pathway, as measured by Western blot, ELISA, cholesterol quantification, and Nile Red staining. These findings suggest that QXTMY may exert protective effects against AS, potentially via suppression of the AGE‐RAGE‐mediated inflammatory axis. However, in vivo validation is still required to support these preliminary conclusions.

## 1. Introduction

Atherosclerosis (AS) is a chronic inflammatory disease of the arterial wall and the primary pathological substrate of most cardiovascular events, including myocardial infarction and stroke [1–3]. Its pathogenesis is multifactorial, involving endothelial dysfunction, subendothelial retention of lipoproteins, infiltration of inflammatory cells, and proliferation of vascular smooth muscle cells (VSMCs) [1, 2]. Among these, macrophages play a central role: they take up modified lipoproteins to become foam cells, secrete proinflammatory cytokines (TNF, IL6, and IL1B), and contribute to plaque progression and instability [4, 5]. VSMCs also contribute significantly to AS; they undergo phenotypic switching from a contractile to a synthetic state, migrate into the intima, produce extracellular matrix, and can also form foam cells, thereby affecting plaque stability [6, 7]. Thus, an ideal antiatherosclerotic agent might target both inflammatory macrophages and abnormal VSMC behavior [8, 9]. However, current standard therapies, primarily statins, antiplatelet drugs, and revascularization procedures, mainly reduce low‐density lipoprotein cholesterol or prevent thrombosis, but they do not fully resolve inflammation or reverse established plaques, and they carry considerable adverse effects [10–13]. Consequently, there is growing interest in multitarget, natural product‐based therapies, particularly from traditional Chinese medicine (TCM).

In clinical practice, several TCM formulas have shown promise for AS, including Xuefu Zhuyu Decoction [14–16], Danshen Decoction [17, 18], and Qingre Huoxue Decoction [19]. Randomized controlled trials and meta‐analyses have provided preliminary evidence that TCM formulas can improve carotid intima‐media thickness, reduce inflammatory markers, and enhance clinical symptoms when used as adjuncts to statins [20]. However, these formulas are predominantly based on the “blood stasis” theory and may not address other TCM syndromes such as heart‐fire hyperactivity or yin deficiency, which are common in patients with AS accompanied by anxiety, palpitations, or insomnia.

Qingxin Tongmai Yin (QXTMY) is a classical TCM formula originally documented by Yaohong Song for cardiovascular conditions. It consists of eight herbs: *Pseudostellariae Radix* (Tai Zi Shen), *Salviae Radix* (Dan Shen), *Lonicerae Japonicae Flos* (Jin Yin Hua), *Paeoniae Rubra Radix* (Chi Shao), *Achyranthis Bidentatae Radix* (Niu Xi), *Nardostachyos Radix* (Gan Song), *Ophiopogonis Radix* (Mai Dong), and *Rehmanniae Radix* (Di Huang). Unlike many blood‐activating formulas, QXTMY is designed to clear heart fire, nourish yin, and resolve toxin, a theoretical advantage for AS patients with inflammatory “heat” or stress‐related symptoms. To date, however, no rigorous clinical trial has evaluated QXTMY for AS, and its pharmacological mechanisms remain largely unexplored. Thus, a systematic investigation of its active components, potential targets, and pathways is urgently needed to guide future clinical studies.

To address this gap, we applied a network pharmacology approach, a computational strategy that integrates drug‐target‐disease networks, to predict the multitarget mechanisms of QXTMY against AS. By constructing compound‐target‐pathway networks, we identified the AGE‐RAGE signaling cascade as a third top‐enriched pathway, with multiple core compounds (quercetin, luteolin, and kaempferol) predicted to interact with inflammatory targets such as TNF, IL6, and NF‐κB. Notably, the Gene Ontology (GO) biological processes most significantly associated with QXTMY were related to response to lipopolysaccharide (LPS), other pathogenic stimuli, and inflammatory response, whereas VSMC‐specific processes (e.g., smooth muscle cell proliferation or contractile function) were not significantly enriched in our initial analysis. Therefore, we chose to focus our in vitro validation on macrophages (THP‐1‐derived) as the primary cell type predicted to mediate QXTMY’s anti‐inflammatory effects. We fully acknowledge that VSMCs are also critical in AS; however, the present study is an exploratory step based on network predictions, and future work will examine VSMC involvement.

In the present study, we aimed to (1) systematically identify the bioactive compounds and putative targets of QXTMY using database mining; (2) construct compound‐target‐disease and protein–protein interaction (PPI) networks; (3) perform GO and Kyoto Encyclopedia of Genes and Genomes (KEGG) pathway enrichment analyses; and (4) experimentally validate the predicted AGE‐RAGE/NF‐κB pathway in THP‐1‐derived macrophages. The results provide a mechanistic basis for the antiatherosclerotic activity of QXTMY and highlight its potential advantages as a multitarget, anti‐inflammatory TCM formula.

## 2. Materials and Methods

### 2.1. Identification and Collection of Bioactive Compounds in QXTMY

The chemical constituents of QXTMY were systematically sourced from two major databases: the Traditional Chinese Medicine Systems Pharmacology Database and Analysis Platform (TCMSP; https://tcmsp-e.com/) and SymMap v2 (http://www.symmap.org/) and the published literature. To prioritize compounds with favorable pharmacokinetic properties, a filtration process was implemented based on oral bioavailability (OB) >30% and a drug‐likeness (DL) score ≥0.18, ensuring that only molecules with potential for adequate bioavailability were retained for further investigation.

### 2.2. Target Identification of QXTMY and AS

Potential protein targets of QXTMY’s bioactive compounds were retrieved from three databases: TCMSP (using the same OB and DL criteria), the Herbal Ingredients’ Targets Platform (HIT 2.0; http://www.badd-cao.net:2345/), and SwissTargetPrediction (STP; http://www.swisstargetprediction.ch/), where predictions with a probability score >0 were included. All targets were compiled into a single list, and duplicate entries (identical gene symbols) were removed to generate the final set of unique targets. No weighting or score‐based prioritization was applied across databases as TCMSP and HIT 2.0 do not provide standardized confidence scores comparable to STP probabilities. All targets were limited to *Homo sapiens*, and compound structures were cross‐referenced in PubChem (https://pubchem.ncbi.nlm.nih.gov/). Gene nomenclature was standardized via the STRING database (https://cn.string-db.org/).

AS‐associated targets were collected by searching the keyword “atherosclerosis” in GeneCards (relevance score ≥5), OMIM (https://omim.org/), the Therapeutic Target Database (TTD; http://db.idrblab.net/ttd/), and DrugBank (https://go.drugbank.com/), retaining only human genes. Overlapping targets between QXTMY and AS were identified using the Bioinformatics & Evolutionary Genomics Venn tool (http://bioinformatics.psb.ugent.be/webtools/Venn/) and further analyzed with Venny 2.1.

### 2.3. PPI Network Construction

A PPI network for the common targets was constructed using the STRING database (v11.5) under a high‐confidence interaction score threshold (≥0.90) for *Homo sapiens*, with disconnected nodes hidden. The network was visualized and analyzed in Cytoscape (v3.9.1). Key hub targets were identified by integrating three topological methods: the CytoNCA plugin for centrality, MCODE for module extraction, and cytoHubba for hub gene ranking. Targets consistently identified across all three methods were considered core targets and visualized via Venny 2.1.

### 2.4. Functional and Pathway Enrichment Analysis

GO and KEGG pathway enrichment analyses for the core targets were conducted using the Metascape platform (https://metascape.org/). The top 15 significantly enriched terms from each category were selected and graphically represented with the bioinformatics online platform (http://www.bioinformatics.com.cn/).

### 2.5. Preparation of QXTMY Decoction

The QXTMY decoction was prepared from the following eight authenticated herbs: *Pseudostellariae Radix* (10 g), *Salviae Radix* (10 g), *Lonicerae Japonicae Flos* (10 g), *Paeoniae Rubra Radix* (10 g), *Achyranthis Bidentatae Radix* (10 g), *Nardostachyos Radix* (10 g), *Ophiopogonis Radix* (10 g), and *Rehmanniae Radix* (10 g), all supplied by Nanjing Hospital of Chinese Medicine Affiliated to Nanjing University of Chinese Medicine. The preparation involved soaking the herbs in 630 mL of distilled water for 30 min, followed by decoction: boiling at 100°C initially and then simmering at 90 ± 2°C for 30 min. The filtrate was collected through a 100‐mesh sieve, and the extraction was repeated under identical conditions. The combined filtrates were concentrated under vacuum (40°C, 0.08 MPa) to a final concentration of 2.5 g of crude herb/mL (determined by freeze‐drying weight). Aliquots were stored at −20°C. They were diluted with culture medium to final working concentrations of 50 μg/mL (low, L), 150 μg/mL (medium, M), and 450 μg/mL (high, H), based on preliminary cell viability assays (Figure S1).

### 2.6. Cell Culture and Differentiation

THP‐1 human monocytes (Shanghai Zhong Qiao Xin Zhou Biotechnology Co., Ltd., Cat. No. ZQ0086) were maintained in RPMI‐1640 medium supplemented with 10% fetal bovine serum at 37°C in a 5% CO_2_ atmosphere. To differentiate into macrophages, cells were exposed to 100 ng/mL phorbol‐12‐myristate‐13‐acetate (PMA; MedChemExpress; Cat. No. HY‐18739) for 48 h. Differentiated macrophages were then stimulated with 100 ng/mL oxidized low‐density lipoprotein (ox‐LDL; MedChemExpress; Cat. No. HY‐NP013) in serum‐free medium for 24 h to induce foam cell formation.

### 2.7. Analysis of Lipid Accumulation via Nile Red Staining

THP‐1‐derived macrophages were incubated with ox‐LDL (100 ng/mL) for 24 h, followed by treatment with QXTMY (50, 150, or 450 μg crude herb/mL) for another 24 h. Cells were then stained with 1 μM Nile Red (MedChemExpress; Cat. No. HY‐D0718) for 10 min in the dark. Lipid droplet accumulation was visualized and imaged using a Leica DM2500 fluorescence microscope.

After ox‐LDL treatment, cells were stained with 1 μM Nile Red (MedChemExpress; Cat. No. HY‐D0718) for 10 min in the dark. Lipid droplet accumulation was visualized and imaged using a Leica DM2500 fluorescence microscope.

### 2.8. Cellular Cholesterol Efflux Assay

Following treatment with QXTMY (50, 150, or 450 μg crude herb/mL) for 24 h, THP‐1‐derived macrophages were washed twice with PBS. Cells were then labeled with [^3^H]‐cholesterol (0.5 μCi/mL; PerkinElmer, Cat. No. NET139001MC) in DMEM containing 0.1% fatty acid‐free BSA for 24 h at 37°C. After labeling, cells were washed three times with PBS to remove unincorporated radioactivity and equilibrated in serum‐free DMEM for 2 h. To measure cholesterol efflux, cells were incubated for 6 h in DMEM containing 0.2% BSA supplemented with human apolipoprotein A‐I (ApoA‐I, 10 μg/mL; Sigma–Aldrich, Cat. No. A0722) and high‐density lipoprotein (HDL, 50 μg/mL; MilliporeSigma, Cat. No. 437641) as cholesterol acceptors. After the efflux period, the medium was collected, and the cells were lysed in 0.1 M NaOH. Radioactivity in the medium and cell lysates was measured by liquid scintillation counting (Tri‐Carb 4910TR, PerkinElmer). Cholesterol efflux was calculated as follows:

% Efflux=medium radioactivity/medium radioactivity+cell lysate radioactivity×100%.

Each condition was performed in triplicate, and the results were normalized to the control group (no QXTMY treatment).

### 2.9. Quantification of Free and Total Cholesterol (TC)

Levels of free cholesterol (FC) and TC in the culture medium were determined using commercial assay kits (Applygen Technologies Inc.; TC: E1005 and FC: E1016) following the manufacturer’s instructions.

### 2.10. Cytokine Measurement by ELISA

The concentrations of TNF‐α (Neobioscience; Cat. NOV‐FM‐E100136), IL‐10 (Neobioscience; Cat. NOV‐NB‐E10155), IL‐6 (Neobioscience; Cat. EHC007) and IL‐1β (Neobioscience; Cat. NOV‐FM‐E100053) in cell culture supernatants were quantified using the respective ELISA kits according to the provided protocols.

### 2.11. Western Blot Analysis

Western blot analysis was conducted following a previously described method [21]. Briefly, proteins (25 μg per sample) were separated by 4%–12% SDS‐polyacrylamide gel electrophoresis (SDS‐PAGE) and transferred onto polyvinylidene fluoride (PVDF) membranes. The membranes were blocked with 5% skim milk for 1 h at room temperature and subsequently incubated overnight at 4°C with the following primary antibodies: RAGE (1:2000; AA1395), phospho‐NF‐kB p65 (1:2000; AP0124), and β‐actin (1:10,000; AC006). All primary and secondary antibodies were purchased from ABclonal Biotechnology Co., Ltd. (Wuhan, China). After incubation with horseradish peroxidase (HRP)‐conjugated secondary antibodies, protein bands were visualized using an enhanced chemiluminescence (ECL) detection kit (PECL08, Proteinbio, China) and quantified with the Gel‐Pro image analysis software (Media Cybernetics, Las Vegas, USA).

### 2.12. Statistical Analysis

All data are expressed as the mean ± SD from a minimum of three independent experiments. Statistical comparisons were performed using GraphPad Prism 10.0 via one‐way ANOVA, followed by Tukey’s post hoc test. A *p*‐value < 0.05 was considered statistically significant.

## 3. Results

### 3.1. Potential Active Components in QXTMY

**Figure 1:**
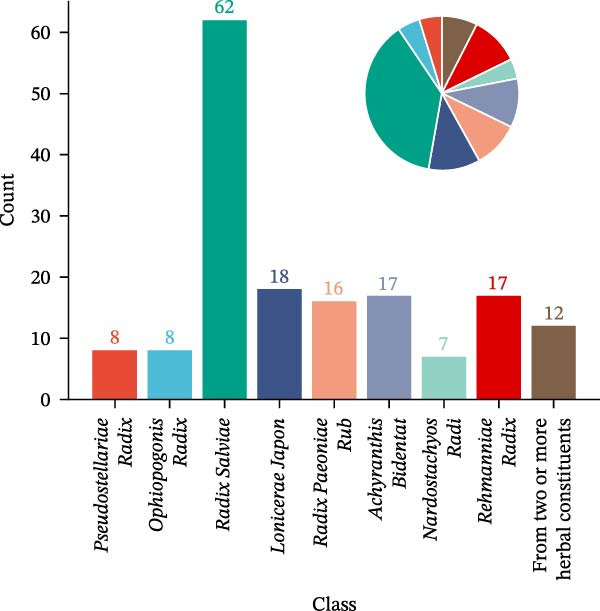
Bar with pie. Potential active components in QXTMY from the TCMSP and SymMap v2 databases.

A total of 165 potential active components were identified, with the distribution as follows: 8 from *Pseudostellariae Radix*, 8 from *Ophiopogonis Radix*, 62 from *Radix Salvia*, 18 from *Lonicerae Japon*, 16 from *Radix Paeoniae Rub*, 17 from *Achyranthis Bidentat*, 7 from *Nardostachyos Radi*, 17 from *Rehmanniae Radix*, and 12 from two or more herbal constituents (Figure 1).

### 3.2. Common Targets of QXTMY and AS

**Figure 2:**
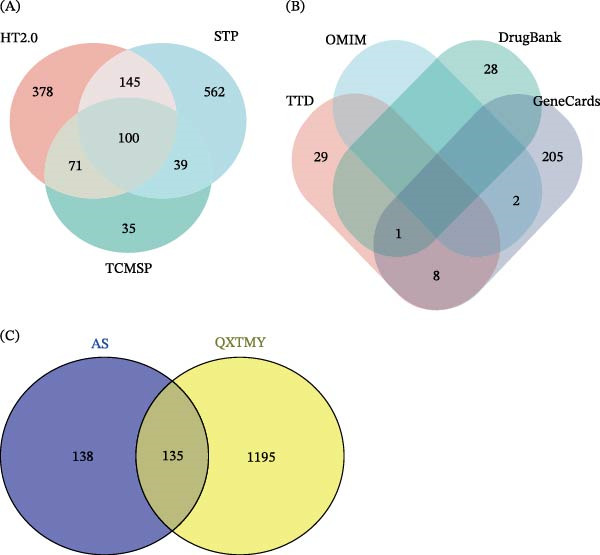
Venn diagrams. (A) Potential targets of QXTMY came from three databases. (B) AS‐related targets came from four databases. (C) The intersection genes of identified AS‐related targets and targets of QXTMY.

A total of 1807 potential targets were predicted for 165 bioactive compounds in QXTMY using TCSMP, HIT2.0, and STP. After removing duplicates and standardizing protein names to gene symbols, 1357 unique targets were retained (Figure 2A). For AS, 323 disease‐related targets were retrieved from four databases: GeneCards (216 targets, relevance score ≥5), TTD (76), DrugBank (29), and OMIM (2). Following duplicate removal, 273 AS‐associated targets were identified (Figure 2B). Intersection analysis via Venny software revealed 135 shared targets between QXTMY and AS (Figure 2C), which were selected for further investigation.

### 3.3. Construction and Analysis of a PPI Network

The 135 potential therapeutic targets of QXTMY against AS were used to construct a PPI network. The resulting network comprised 134 nodes and 2485 edges, with an average node degree of 9.84 (Figure 3A). To identify the core PPI network, the initial network was visualized using Cytoscape 3.9.1 (Figure 3B). Protein clustering analysis performed with the MCODE plugin revealed five distinct clusters (Figure 3C). Among these, Cluster 1 exhibited the highest score (40.227) and consisted of 45 nodes and 885 edges, making it the focus for subsequent core node analysis. Further topological evaluation of the PPI network was conducted using the CytoNCA plugin, which calculated key parameters for each node. To refine the core network, stringent topological filters were applied, including betweenness centrality (BC > 164.6418), closeness centrality (CC > 0.6426), and degree centrality (DC > 59). This screening yielded a core PPI network containing 6 nodes and 14 edges (Figure 3D). Additionally, the top 10 hub genes (TNF, IL6, IL1B, ALB, CCL2, ICAM1, TLR4, insulin (INS), CXCL8, and MMP9) were identified using the MCC algorithm in the cytoHubba plugin (Figure 3E). Finally, intersection analysis via Venny 2.1 revealed seven common core nodes shared by cytoHubba, MCODE, and CytoNCA, which were selected for further investigation (Figure 3F).

Figure 3PPI network of QXTMY‐AS. (A) The interactive PPI network obtained from the STRING database with species limited to “*Homo sapiens*,” the minimum required interaction score set to 0.98, and the independent target protein nodes hidden. (B) Original PPI network from the STRING database imported to Cytoscape 3.9.1 to obtain a new network. It contains 134 nodes and 2485 edges. (C) Core PPI network screened from (B) in Cytoscape 3.9.1 using the MCODE plugin. (D) Core PPI network screened from (B) in Cytoscape 3.9.1 using the CytoNCA plugin. (E) Core PPI network screened from (B) in Cytoscape 3.9.1 using the MCC algorithm in the cytoHubba plugin. (F) The intersection core nodes shared by cytoHubba, MCODE, and CytoNCA.

**Figure:**
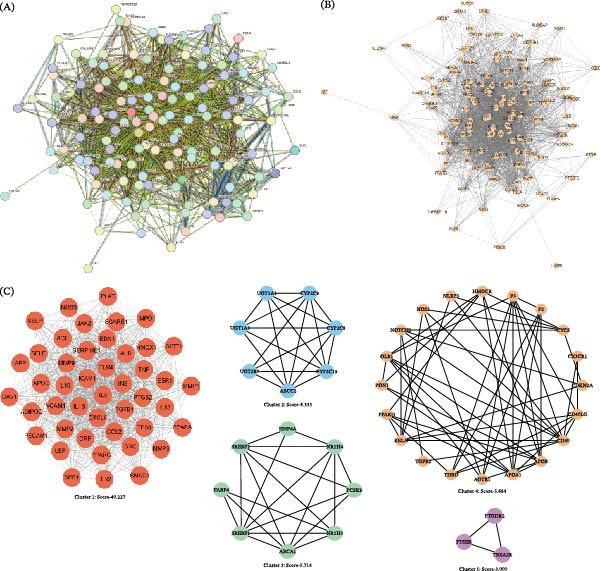

**Figure:**
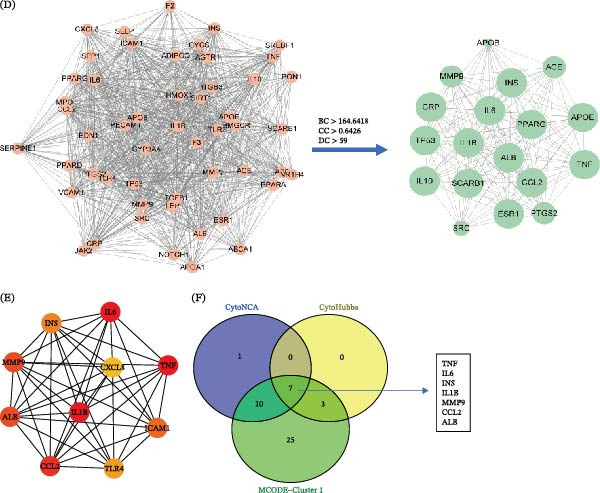

### 3.4. GO and KEGG Pathway Enrichment Analysis

**Figure 4:**
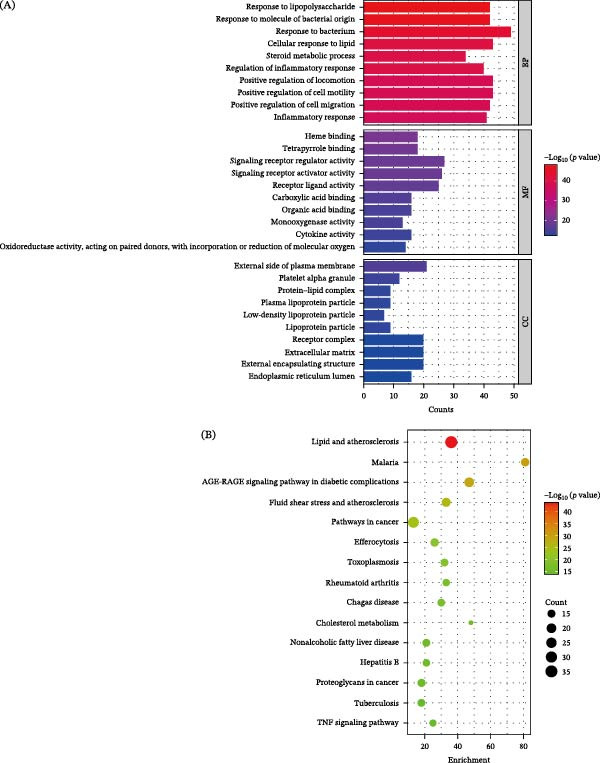
A total of 135 selected targets were subjected to GO enrichment analysis and KEGG pathway analysis. (A) The GO enrichment analysis bar diagram. (B) The KEGG pathway analysis bubble diagram.

To investigate the functional roles of the 135 overlapping targets, GO and KEGG pathway enrichment analyses were performed. GO analysis indicated that QXTMY targets are primarily involved in biological processes such as the cellular response to LPS, bacterial‐derived molecules, and other pathogenic stimuli (Figure 4A). Cellular component enrichment was observed for the external side of the plasma membrane, the platelet alpha granule lumen, and plasma lipoprotein particles (Figure 4A). Molecular function analysis revealed significant associations with heme binding, tetrapyrrole binding, and signaling receptor regulator activity (Figure 4A).

**Figure 5:**
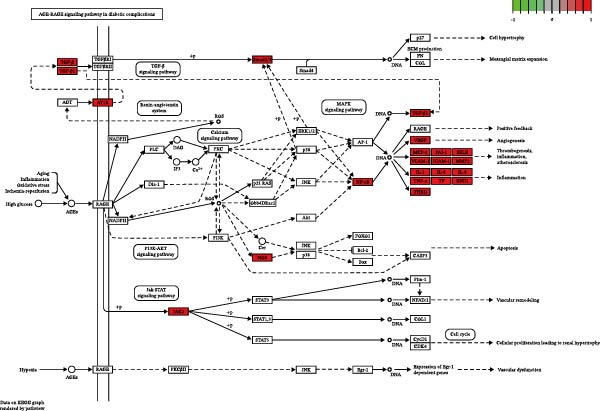
The map of the AGE‐RAGE signaling pathway. There are 21 target genes (red color) enriched in the AGE‐RAGE signaling pathway.

KEGG pathway analysis highlighted “Lipid and atherosclerosis,” “Malaria,” and the “AGE‐RAGE signaling pathway in diabetic complications” as the most significantly enriched pathways (Figure 4B and Table 1). The AGE‐RAGE signaling pathway was particularly notable, corroborating previous studies linking this pathway to AS pathogenesis [22]. A schematic of this pathway is depicted in Figure 5. Based on these findings and supporting evidence, we hypothesized that the AGE‐RAGE signaling pathway is a pivotal mechanism through which QXTMY exerts its antiatherosclerotic action.

**Table 1:** Top 15 pathways of KEGG enrichment analysis.

Term	−Log_10_ (*p*‐value)	Gene count
hsa05417: lipid and atherosclerosis	−44	35
hsa05144: malaria	−30	18
hsa04933: AGE‐RAGE signaling pathway in diabetic complications	−29	21
hsa05418: fluid shear stress and atherosclerosis	−26	21
hsa05200: pathways in cancer	−24	30
hsa04148: efferocytosis	−20	18
hsa05145: toxoplasmosis	−19	16
hsa05323: rheumatoid arthritis	−17	14
hsa05142: Chagas disease	−17	14
hsa04979: cholesterol metabolism	−15	11
hsa04932: nonalcoholic fatty liver disease	−15	15
hsa05161: hepatitis B	−15	15
hsa05205: proteoglycans in cancer	−15	16
hsa05152: tuberculosis	−14	15
hsa04668: TNF signaling pathway	−14	13

### 3.5. Prediction of QXTMY Components Targeting the AGE‐RAGE Pathway

**Figure 6:**
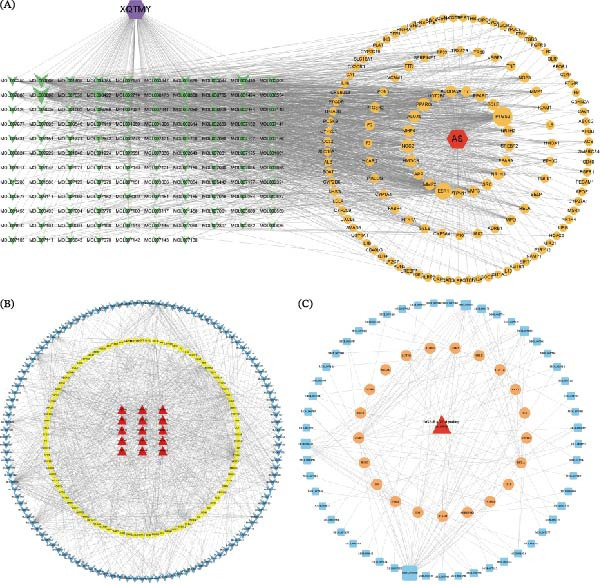
(A) The compound‐disease target network was constructed by Cytoscape 3.9.1. The green nodes represent the compounds in QXTMY, and the yellow nodes represent the targets of QXTMY on AS. (B) The compound‐target‐pathway network. The yellow circle nodes represent targets, the red triangle nodes represent the related pathways. The blue nodes represent the compounds. (C) The compound‐target network in the AGE‐RAGE signaling pathway. The yellow circle nodes represent targets, the red triangle nodes represent the AGE‐RAGE signaling pathway. The blue nodes represent the compounds.

Compound‐disease‐target (C–D–T) and compound‐target‐pathway (C–T–P) networks were established using Cytoscape (Figure 6A,B). Network topology analysis identified quercetin as the most connected compound, followed by luteolin, stigmasterol, kaempferol, and β‐sitosterol (Degree > 100). Furthermore, quercetin, kaempferol, luteolin, and cryptotanshinone (Degree > 10) were predicted as key anti‐AS constituents, potentially mediating their therapeutic effects by influencing the AGE‐RAGE signaling pathway (Figure 6C).

### 3.6. QXTMY Attenuates ox‐LDL‐Induced Lipid Accumulation and Inflammation in THP‐1 Cells

**Figure 7:**
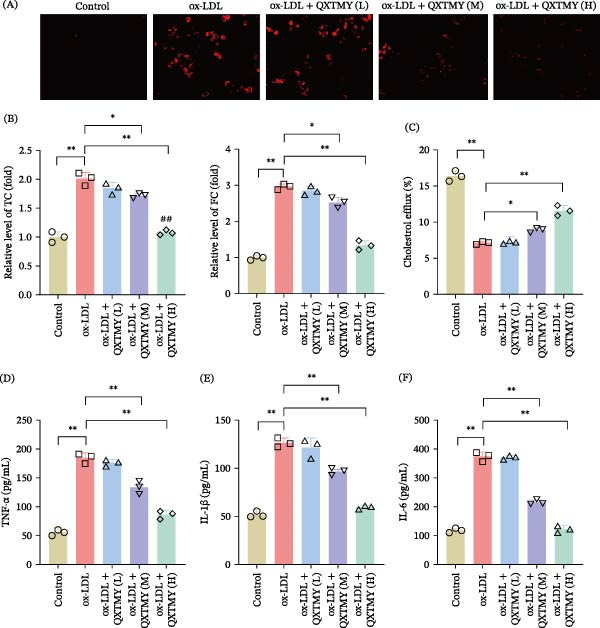
Effect of QXTMY on lipid accumulation and secretory levels of inflammatory factors in ox‐LDL‐induced THP‐1 cells. (A) Nile Red staining of THP‐1 cells. Scale bar = 100 μm. (B) Total cholesterol (TC) and free cholesterol (FC) levels measured using corresponding commercial kits. (C) Cellular cholesterol efflux assessed by liquid scintillation counting of cell‐associated [^3^H]‐cholesterol. (D–F) Effect of QXTMY on the secretory levels of TNF‐α, IL‐1β, and IL‐6, respectively. Data are shown as mean ± SD (*n* = 3).  ^∗^ *p* < 0.05,  ^∗∗^ *p* < 0.01. L, M, and H denote 50 μg/mL, 150 μg/mL, and 450 μg/mL, respectively. One‐way ANOVA and Tukey’s test.

Macrophage foam cell formation, driven by intracellular lipid accumulation and enhanced inflammatory cytokine secretion, is a hallmark of AS [5, 23]. Nile Red staining revealed that treatment with 100 ng/mL ox‐LDL significantly promoted lipid deposition in THP‐1‐derived macrophages, confirming successful foam cell generation. QXTMY administration dose‐dependently suppressed lipid accumulation (Figure 7A). Consistent with this, ox‐LDL elevated intracellular levels of TC and FC, effects that were significantly reversed by QXTMY (Figure 7B). Moreover, QXTMY treatment ameliorated the ox‐LDL‐impaired cellular cholesterol efflux capacity (Figure 7C). These results collectively demonstrate that QXTMY inhibits ox‐LDL‐induced foam cell formation.

The anti‐inflammatory effect of QXTMY was evaluated by measuring the secretion of proinflammatory cytokines (TNF‐α, IL‐1β, and IL‐6) via ELISA. Ox‐LDL stimulation significantly increased the release of these cytokines, which was markedly attenuated by pretreatment with QXTMY (Figure 7D–F). These data indicate that QXTMY effectively counteracts both lipid accumulation and inflammatory responses triggered by ox‐LDL in THP‐1 macrophages.

### 3.7. QXTMY Suppresses ox‐LDL‐Induced Activation of the AGE‐RAGE Pathway in THP‐1 Cells

**Figure 8:**
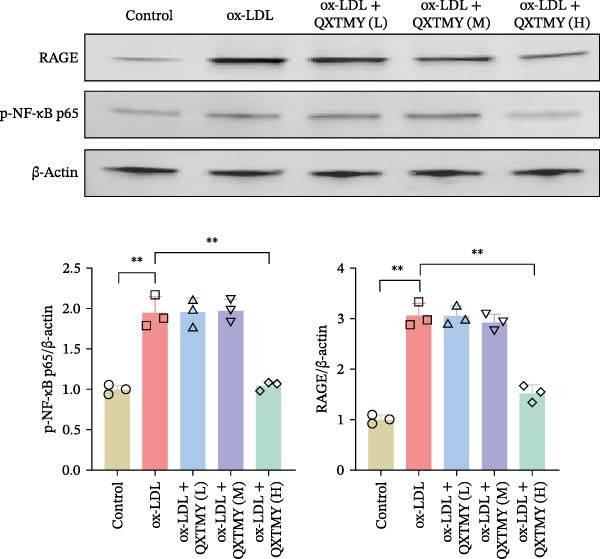
Effect of QXTMY on protein levels of the AGE‐RAGE‐NF‐κB signaling pathway in ox‐LDL‐induced THP‐1 cells. Representative immunoblots of RAGE and phospho (p)‐NF‐κB p65, and densitometric quantification of the corresponding proteins. Data are shown as mean ± SD (*n* = 3).  ^∗∗^ *p* < 0.01. L, M, and H denote 50 μg/mL, 150 μg/mL, and 450 μg/mL, respectively. One‐way ANOVA and Tukey’s test.

Guided by KEGG analysis and literature evidence, we investigated the AGE‐RAGE signaling pathway as a potential target of QXTMY. Western blot analysis demonstrated that ox‐LDL stimulation significantly increased the protein expression of RAGE compared to the control group (*p* < 0.05). QXTMY treatment effectively inhibited this ox‐LDL‐induced RAGE upregulation (*p* < 0.05, Figure 8). Since NF‐κB is a major downstream effector of RAGE signaling, we assessed its phosphorylation status. QXTMY significantly reduced the phosphorylation level of NF‐κB p65 at Ser536 compared to the ox‐LDL‐treated group (*p* < 0.05, Figure 8). These findings suggest that QXTMY mitigates AS‐associated inflammation by inhibiting the AGE‐RAGE‐NF‐κB signaling cascade.

## 4. Discussion

AS is a chronic, multifactorial inflammatory disease of the arterial wall, for which current pharmacotherapies, primarily statins, antiplatelet agents, and antihypertensives, mainly target individual risk factors [24]. Despite their efficacy, a substantial residual risk of cardiovascular events persists, largely driven by ongoing inflammation, plaque vulnerability [25], and poor patient adherence due to adverse effects [26]. TCM formulations such as QXTMY offer a complementary paradigm: multicomponent, multitarget regulation that may address the complex pathophysiology of AS more holistically. In this study, using network pharmacology and in vitro validation, we identified 128 bioactive components of QXTMY that interact with 135 AS‐related targets, with quercetin, luteolin, kaempferol, and β‐sitosterol as core constituents. Beyond confirming known mechanisms, our results reveal distinct clinical advantages of QXTMY as a compound decoction compared to conventional therapies or single isolated compounds.

Unlike statins, which primarily lower LDL cholesterol [27], QXTMY concurrently downregulates proinflammatory cytokines (TNF, IL6, and IL1B), CCL2, and MMP9, all of which are independent predictors of plaque rupture and cardiovascular events [28, 29]. This coordinated action suggests that QXTMY could address the “residual inflammatory risk” that remains even after optimal lipid lowering, a major unmet clinical need [30, 31]. Moreover, the regulation of AGE‐RAGE signaling, which we experimentally confirmed by reduced RAGE expression and NF‐κB inhibition in THP‐1 macrophages, directly targets a key driver of vascular inflammation, particularly in diabetic patients, a population at markedly higher AS risk.

Beyond inflammation, our KEGG analysis also strongly implicated the “Lipid and atherosclerosis” pathway (top rank). This pathway encompasses multiple processes central to AS, including cholesterol uptake, efflux, and foam cell formation. In our in vitro experiments, QXTMY treatment significantly reduced lipid accumulation (Nile Red staining) and increased cholesterol efflux (Figure 7), supporting a direct effect on macrophage foam cell formation. While we focused on the AGE‐RAGE axis, the antifoam cell effect may also involve regulation of scavenger receptors (e.g., CD36 and SR‐A) or cholesterol transporters (ABCA1 and ABCG1), a possibility that warrants future investigation. Another enriched pathway, “Fluid shear stress and atherosclerosis,” was also identified (−Log_10_ [*p*‐value] = 7.6). Although not experimentally validated here, this pathway links mechanical forces to endothelial dysfunction and plaque localization [32, 33]. Whether QXTMY influences shear‐sensitive genes (e.g., KLF2 and eNOS) remains unknown and could be explored in endothelial cell models. Finally, the “Malaria” pathway (ranked second) may seem unrelated, but it shares key inflammatory mediators (e.g., TNF, IL6, and CCL2) with AS. This overlap further supports the anti‐inflammatory potential of QXTMY rather than a direct antimalarial effect.

While individual compounds such as quercetin and luteolin have known antiatherosclerotic effects, QXTMY provides them in naturally balanced ratios, allowing additive or synergistic effects without the toxicity risks of high‐dose monotherapy. For example, quercetin and kaempferol together may more effectively suppress macrophage foam cell formation through both the Piezo1/MAPK/NF‐κB and HO‐1/Nrf2 pathways [34, 35], while β‐sitosterol complements these effects by improving endothelial survival and dyslipidemia [36]. This synergy is a core clinical advantage: it enables lower effective doses of each component, reduces the likelihood of adverse reactions (e.g., statin‐associated myopathy or gastrointestinal bleeding from antiplatelets), and thereby enhances long‐term patient compliance, a critical factor in chronic AS management.

Among the core active constituents identified by network analysis, cryptotanshinone, a diterpenoid from *Salvia miltiorrhiza* (Dan Shen), had a DC > 10. Cryptotanshinone was previously reported to suppress macrophage inflammation via the inhibition of NF‐κB and MAPK pathways [37, 38]. In our compound‐target network, cryptotanshinone was predicted to interact with TNF, IL6, and RAGE, consistent with its known anti‐inflammatory properties. Although our in vitro experiments used the whole QXTMY decoction rather than isolated compounds, the presence of cryptotanshinone likely contributes to the observed inhibition of the AGE‐RAGE/NF‐κB axis. Future fractionation studies could quantify its individual contribution.

Our network analysis identified TNF, IL6, MMP9, and RAGE as central targets. Clinically, elevated serum levels of these markers correlate with plaque burden, instability, and poor prognosis [39–41]. Therefore, QXTMY may be particularly beneficial for AS patients with high inflammatory phenotypes (e.g., elevated hs‐CRP, IL6, or MMP9) or those with concomitant diabetes/INS resistance, where AGE‐RAGE activation is prominent. Future clinical trials could use these biomarkers to stratify patients, moving toward a personalized TCM approach, a distinct advantage over the current “one‐size‐fits‐all” strategy.

Although formal toxicological studies are still needed, the natural origin of QXTMY constituents and their long history of use in TCM suggest a potentially lower incidence of severe adverse effects compared to chronic statin or antiplatelet therapy. This is particularly important for elderly AS patients, who often require polypharmacy and are vulnerable to drug–drug interactions. The decoction form also allows flexible dose titration, an advantage over fixed‐dose combination pills.

Several limitations of this study should be noted. First, the current network still primarily reflects database‐ and literature‐derived information and cannot fully represent the hierarchical structure of the formula. Future studies should integrate quantitative chemical profiling (e.g., HPLC‐MS/MS) with bioactivity‐guided fractionation to better align the network with the Jun‐Chen‐Zuo‐Shi principle. Second, our in vitro validation focused exclusively on the AGE‐RAGE pathway. The roles of lipid metabolism, fluid shear stress, and other enriched pathways were not experimentally tested, and conclusions regarding these mechanisms remain speculative based on network predictions alone. Third, while our data suggest that QXTMY modulates the AGE‐RAGE pathway, further mechanistic studies are required to fully delineate this relationship. Fourth, the absence of in vivo validation represents an important constraint, and future work should include animal models of AS to confirm these findings. Fifth, no positive control drug (e.g., a statin or a specific RAGE inhibitor such as FPS‐ZM1) was included in our cell‐based assays; therefore, the relative efficacy of QXTMY compared to standard therapies cannot be assessed. Future studies should incorporate appropriate positive controls to benchmark the anti‐inflammatory and antiatherosclerotic effects of QXTMY. Finally, the mechanistic validation was limited to Western blot analysis of RAGE and NF‐κB in a single cell line (THP‐1‐derived macrophages). While this supports the network‐predicted AGE‐RAGE pathway, it does not constitute a comprehensive mechanistic dissection. Additional approaches, such as target‐specific knockdown, pharmacological inhibitors, or reporter gene assays, are required to firmly establish causality.

## 5. Conclusion

In conclusion, this study demonstrates that QXTMY exerts antiatherosclerotic effects through a multitarget, multipathway mechanism, with the AGE‐RAGE/NF‐κB signaling axis identified as a central regulatory node. The consistency between our network pharmacology predictions and the preliminary in vitro data obtained in THP‐1 macrophages provides initial experimental support for this mechanistic framework. These findings offer a valuable foundation for understanding the pharmacological basis of QXTMY.

**Nomenclature**
QXTMY: Qingxin Tongmai Yin decoction
AS: Atherosclerosis
PPI: Protein–protein interaction
OB: Oral bioavailability
DL: Drug‐likeness
AGE: Advanced glycation end products
BC: Betweenness centrality
CC: Closeness centrality
CCL2: C–C motif chemokine 2
CXCL: C–X–C motif chemokine ligand
GO: Gene Ontology
KEGG: Kyoto Encyclopedia of Genes and Genomes
MMP: Matrix metallopeptidase
DC: Degree centrality
ECs: Endothelial cells
VSMC: Vascular smooth muscle cell
PAI‐1: Plasminogen activator inhibitor‐1
ET‐1: Endothelin‐1
INS: Insulin.

## Author Contributions

**Yangfan Huang, Zhengshu Xia, Zhiyong Yu, and Jiaying Song:** investigation, data curation, validation, writing – original draft. **Jiaqian Fang**: investigation, data curation, validation. **Yaohong Song**: conceptualization, supervision, writing – review and editing. **Rui Chen**: writing – original draft, conceptualization, funding acquisition, project administration, supervision, writing – review and editing.

## Funding

This study was supported by the Research Project on Traditional Chinese Medicine Preparations in Medical Institutions of Nanjing City (Grant NJCC‐ZJ‐202415), the Scientific Research Projects on Traditional Chinese medicine and integrated traditional Chinese and Western medicine in Jiangsu Province (Grant CYTF2026038), the Nanjing Health Science and Technology Development Special Fund Project for 2024 (Grant YKK24171), and the Song Yaohong Nanjing Famous Chinese Medicine Practitioner’s Studio (Grant 2023‐NJSMZYGZS‐SYH).

## Conflicts of Interest

The authors declare no conflicts of interest.

## Supporting Information

Additional supporting information can be found online in the Supporting Information section.

## Supporting information


**Supporting Information** Figure S1: Effect of QXTMY on the viability of ox‐LDL‐induced THP‐1 cells.

## Data Availability

The data that support the findings of this study are available from the corresponding author (Rui Chen) upon reasonable request.

## References

[1] Xing Y. and Lin X. , Challenges and Advances in the Management of Inflammation in Atherosclerosis, Journal of Advanced Research. (2025) 71, 317–335, 10.1016/j.jare.2024.06.016. 38909884 PMC12126742

[2] Ait-Oufella H. and Libby P. , Inflammation and Atherosclerosis: Prospects for Clinical Trials, Arteriosclerosis, Thrombosis, and Vascular Biology. (2024) 44, no. 9, 1899–1905, 10.1161/ATVBAHA.124.320155. 39167675 PMC11343092

[3] Tasouli-Drakou V. , Ogurek I. , Shaikh T. , Ringor M. , DiCaro M. V. , and Lei K. C. , Atherosclerosis: A Comprehensive Review of Molecular Factors and Mechanisms, International Journal of Molecular Sciences. (2025) 26, no. 3, 10.3390/ijms26031364, 1364. 39941130 PMC11818631

[4] Gianopoulos I. and Daskalopoulou S. S. , Macrophage Profiling in Atherosclerosis: Understanding the Unstable Plaque, Basic Research in Cardiology. (2024) 119, no. 1, 35–56, 10.1007/s00395-023-01023-z. 38244055

[5] Wu J. , He S. , and Song Z. , et al.Macrophage Polarization States in Atherosclerosis, Frontiers in Immunology. (2023) 14, 10.3389/fimmu.2023.1185587, 1185587. 37207214 PMC10189114

[6] Fu Z. , Yang S. , Chang X. , Liu P. , and Wang Y. , Vascular Smooth Muscle Cell Metabolic Reprogramming and Phenotypic Remodeling in Atherosclerosis, Cell Death Discovery. (2025) 12, no. 1, 10.1038/s41420-025-02932-9, 64. 41469416 PMC12847746

[7] Azar P. , Jarr K.-U. , Gomez D. , Jørgensen H. F. , Leeper N. J. , and Bochaton-Piallat M.-L. , Smooth Muscle Cells in Atherosclerosis: Essential but Overlooked Translational Perspectives, European Heart Journal. (2025) 46, no. 45, 4862–4875, 10.1093/eurheartj/ehaf337. 40574648 PMC12972687

[8] Wang J. , Wu Q. , and Wang X. , et al.Targeting Macrophage Phenotypes and Metabolism as Novel Therapeutic Approaches in Atherosclerosis and Related Cardiovascular Diseases, Current Atherosclerosis Reports. (2024) 26, no. 10, 573–588, 10.1007/s11883-024-01229-z. 39133247 PMC11392985

[9] Qin X. , Zhou Z. , Zheng Z. , Wen X. , and Yang H. , Targeting Vascular Smooth Muscle Cells With Natural Compounds to Combat Atherosclerosis, Chinese Journal of Natural Medicines. (2026) 24, no. 7, 791–806, 10.1016/S1875-5364(26)61117-X. 42285685

[10] Waksman R. , Merdler I. , Case B. C. , Waksman O. , and Porto I. , Targeting Inflammation in Atherosclerosis: Overview, Strategy and Directions, EuroIntervention. (2024) 20, no. 1, 32–44, 10.4244/EIJ-D-23-00606. 38165117 PMC10756224

[11] Tu Z. , Wei Y. , Liao Y. , Wan Z. , Zhou Z. , and Guo J. , Pathological Mechanisms and Research Progress of Multilevel Intervention Therapy for Coronary Heart Disease, Frontiers in Immunology. (2026) 17, 10.3389/fimmu.2026.1816199, 1816199. 42051526 PMC13110956

[12] Lu W. J. , Li J. Y. , and Shih T. L. , et al.A Naphthalimide Derivative Exerts Potent Antiplatelet and Antithrombotic Activities Without a Bleeding Tendency, Frontiers in Pharmacology. (2025) 16, 10.3389/fphar.2025.1541255, 1541255. 40630131 PMC12234328

[13] Muroke V. and Tardif J.-C. , Inflammation Mediates Residual Cardiovascular Risk in Statin-Treated Patients With Coronary Disease, European Journal of Preventive Cardiology. (2026) 33, no. 5, 752–753, 10.1093/eurjpc/zwaf258. 40269517

[14] Zhu Y. , Chen Y. , and Tong R. , et al.Xuefu Zhuyu Decoction Ameliorates Atherosclerosis in ApoE(−/−) Mice by Regulating Cholesterol Metabolism and Inflammation, Journal of Chromatography B. (2025) 1263, 10.1016/j.jchromb.2025.124693, 124693. 40482366

[15] Jia D. , Zhao M. , and Zhang X. , et al.Transcriptomic Analysis Reveals the Critical Role of Chemokine Signaling in the Anti-Atherosclerosis Effect of Xuefu Zhuyu Decoction, Journal of Ethnopharmacology. (2024) 332, 10.1016/j.jep.2024.118245, 118245. 38679399

[16] Yuan J. , Yan F. , Li W. , and Yuan G. , Network Pharmacological Analysis of Xuefu Zhuyu Decoction in the Treatment of Atherosclerosis, Frontiers in Pharmacology. (2022) 13, 10.3389/fphar.2022.1069704, 1069704. 36532728 PMC9755496

[17] Song Y. , Zhao W. , and Zhang L. , et al.Compound Danshen Decoction Identifies Its Mechanism of Antagonizing Atherosclerosis by Network Pharmacology and Transcriptomics, Naunyn-Schmiedeberg’s Archives of Pharmacology. (2026) 399, no. 6, 7913–7931, 10.1007/s00210-025-04640-8. 41152614

[18] Guo X. , Meng P. , and Zhang L. , et al.Danshen Decoction Stabilizes Vulnerable Atherosclerotic Plaques by Maturating Intraplaque Neovessels via the Piezo1/Yes-Associated Protein/Angiopoietin-1 Pathway, Journal of Ethnopharmacology. (2026) 368, 10.1016/j.jep.2026.121797, 121797. 42081953

[19] He W. , Zhao H. , and Xue W. , et al.Qingre Huoxue Decoction Alleviates Atherosclerosis by Regulating Macrophage Polarization Through Exosomal miR-26a-5p, Drug Design, Development and Therapy. (2024) 18, 6389–6411, 10.2147/DDDT.S487476. 39749190 PMC11693966

[20] Liu W. , Quan S. , and Lin Q. , et al.Systematic Review and Meta-Analysis of Tongxinluo Capsules Combined With Statins in the Treatment of Carotid Atherosclerosis, Phytomedicine. (2026) 154, 10.1016/j.phymed.2026.158056, 158056. 41831382

[21] Chen R. , Fang J. , Sun H. , Yu Z. , Huang Y. , and Song Y. , Calycosin-7-Glucoside Alleviates Atherosclerosis by Inhibiting Ox-LDL-Induced Foam Cell Formation and Inflammatory Response in THP-1-Derived Macrophages via ATF-1 Activation Through the p38/MAPK Pathway, Journal of Inflammation Research. (2025) 18, 5573–5586, 10.2147/JIR.S516160. 40303003 PMC12039851

[22] Wang B. , Jiang T. , and Qi Y. , et al.AGE-RAGE Axis and Cardiovascular Diseases: Pathophysiologic Mechanisms and Prospects for Clinical Applications, Cardiovascular Drugs and Therapy. (2024) 39, no. 6, 1489–1506, 10.1007/s10557-024-07639-0. 39499399 PMC12717123

[23] He Y. and Liu T. , Oxidized Low-Density Lipoprotein Regulates Macrophage Polarization in Atherosclerosis, International Immunopharmacology. (2023) 120, 10.1016/j.intimp.2023.110338, 110338. 37210916

[24] Galli M. , Abbate A. , and Bonaca M. P. , et al.Residual Cardiovascular Risk in Coronary Artery Disease: From Pathophysiology to Established and Novel Therapies, Nature Reviews Cardiology. (2026) 23, no. 7, 500–523, 10.1038/s41569-026-01249-z. 41577834

[25] Arnold N. and Koenig W. , Inflammation in Atherosclerotic Cardiovascular Disease: From Diagnosis to Treatment, European Journal of Clinical Investigation. (2025) 55, no. 7, 10.1111/eci.70020. PMC12169090 40055964

[26] Insani W. N. , Wei L. , and Abdulah R. , et al.Exploring the Association of Adverse Drug Reactions With Medication Adherence and Quality of Life Among Hypertensive Patients: A Cross-Sectional Study, International Journal of Clinical Pharmacy. (2025) 47, no. 2, 354–364, 10.1007/s11096-024-01832-9. 39607658 PMC11919996

[27] Lee Y. J. , Hong B. K. , and Yun K. H. , et al.Alternative LDL Cholesterol–Lowering Strategy vs High-Intensity Statins in Atherosclerotic Cardiovascular Disease: A Systematic Review and Individual Patient Data Meta-Analysis, JAMA Cardiology. (2025) 10, no. 2, 137–144, 10.1001/jamacardio.2024.3911. 39565634 PMC11579890

[28] Kim B. Y. , Son Y. , and Kim B. J. , et al.Atheroma-Relevant 7-Oxysterols Differentially Upregulate Cd14 Expression, International Journal of Molecular Sciences. (2023) 24, no. 13, 10.3390/ijms241310542, 10542. 37445719 PMC10341412

[29] Yao G. , Luan T. , and Song D. , et al.CXCL16 Promotes Macrophage-Driven Inflammation and Vascular Smooth Muscle Cell Phenotypic Switching During Carotid Plaque Destabilization, International Immunopharmacology. (2026) 182, 10.1016/j.intimp.2026.116848, 116848. 42143937

[30] Yu Y. , Cui R. , and He X. , et al.Residual Inflammatory Risk and Intracranial Atherosclerosis Plaque Vulnerability: Insights From High-Resolution Magnetic Resonance Imaging, Journal of Stroke. (2025) 27, no. 2, 207–216, 10.5853/jos.2024.03251. 40494579 PMC12152451

[31] Ridker P. M. , Targeting Residual Inflammatory Risk: The Next Frontier for Atherosclerosis Treatment and Prevention, Vascular Pharmacology. (2023) 153, 10.1016/j.vph.2023.107238, 107238. 37871757

[32] Cheng H. , Zhong W. , and Wang L. , et al.Effects of Shear Stress on Vascular Endothelial Functions in Atherosclerosis and Potential Therapeutic Approaches, Biomedicine & Pharmacotherapy. (2023) 158, 10.1016/j.biopha.2022.114198, 114198. 36916427

[33] Wang W.-L. , Shih Y.-T. , Wei S.-Y. , and Chiu J.-J. , Impacts of Aging and Fluid Shear Stress on Vascular Endothelial Metabolism and Atherosclerosis Development, Journal of Biomedical Science. (2025) 32, no. 1, 10.1186/s12929-025-01177-z, 83. 40890841 PMC12403445

[34] Zhao J. , Ling L. , and Zhu W. , et al.M1/M2 Re-Polarization of Kaempferol Biomimetic NPs in Anti-Inflammatory Therapy of Atherosclerosis, Journal of Controlled Release. (2023) 353, 1068–1083, 10.1016/j.jconrel.2022.12.041. 36549391

[35] Chu T. , Wang Y. , and Wang S. , et al.Kaempferol Regulating Macrophage Foaming and Atherosclerosis Through Piezo1-Mediated MAPK/NF-κB and Nrf2/HO-1 Signaling Pathway, Journal of Advanced Research. (2025) 75, 635–650, 10.1016/j.jare.2024.11.016. 39561922 PMC12789723

[36] Jiang Y.-H. , Li X. , Niu W. , Wang D. L. , Wu B. , and Yang C.-H. , β-Sitosterol Regulated microRNAs in Endothelial Cells Against an Oxidized Low-Density Lipoprotein, Food & Function. (2020) 11, no. 2, 1881–1890, 10.1039/C9FO01976F. 32068754

[37] Li X.-X. , Zheng X. , and Liu Z. , et al.Cryptotanshinone From Salvia Miltiorrhiza Bunge (Danshen) Inhibited Inflammatory Responses via TLR4/MyD88 Signaling Pathway, Chinese Medicine. (2020) 15, no. 1, 10.1186/s13020-020-00303-3, 20. 32158495 PMC7053069

[38] Tang S. , Shen X. Y. , and Huang H. Q. , et al.Cryptotanshinone Suppressed Inflammatory Cytokines Secretion in RAW264.7 Macrophages Through Inhibition of the NF-κB and MAPK Signaling Pathways, Inflammation. (2011) 34, no. 2, 111–118, 10.1007/s10753-010-9214-3. 20490642

[39] Luo Y. , Li X. , Liu Y. , and Wang X. , The Relationship Between Inflammatory Factors and Major Adverse Cardiovascular Events in Atherosclerosis Coronary Artery Ectasia: A Prospective, Cohort Study, Acta Cardiologica. (2025) 80, no. 5, 506–513, 10.1080/00015385.2025.2515306. 40476575

[40] Han Y. , Mao X. , and Wang L. , et al.Increased Levels of Soluble Cluster of Differentiation 40 Ligand, Matrix Metalloproteinase 9, and Matrix Metalloproteinase 2 Are Associated With Carotid Plaque Vulnerability in Patients With Ischemic Cerebrovascular Disease, World Neurosurgery. (2017) 105, 709–713, 10.1016/j.wneu.2017.06.074. 28642174

[41] Grauen Larsen H. , Marinkovic G. , and Nilsson P. M. , et al.High Plasma sRAGE (Soluble Receptor for Advanced Glycation End Products) Is Associated With Slower Carotid Intima-Media Thickness Progression and Lower Risk for First-Time Coronary Events and Mortality, Arteriosclerosis, Thrombosis, and Vascular Biology. (2019) 39, no. 5, 925–933, 10.1161/ATVBAHA.118.312319. 30917679

