# Supplementary material for: Integrating Network Pharmacology and In Vitro Experiments to Elucidate the Antiatherosclerotic Mechanisms of Qingxin Tongmai Yin
Source: Mediators Inflamm. 2026 Aug 5;2026:9752147. doi: 10.1155/mi/9752147 (PMC13439637; doi:10.1155/mi/9752147)
Supplement: Supplementary file 1 [file MI-2026-9752147-s001.docx]

**Figure S1. Effect of QXTMY on the viability of ox-LDL-induced THP-1 cells.** THP-1 cells were stimulated with 100 ng/mL ox-LDL for 24 h and then treated with the indicated concentrations of QXTMY for an additional 24 h. Cell viability was determined using the CCK-8 assay. Data are presented as the mean ± SD (n = 6)
